# Levels of systemic inflammation response index are correlated with tumor-associated bacteria in colorectal cancer

**DOI:** 10.1038/s41419-023-05602-9

**Published:** 2023-01-30

**Authors:** Yinghao Cao, Xin Zheng, Yugang Hu, Jiahuan Li, Binglu Huang, Ning Zhao, Tao Liu, Kailin Cai, Shan Tian

**Affiliations:** 1 Department of Digestive Surgical Oncology, Cancer Center, Union Hospital, Tongji Medical College, Huazhong University of Science and Technology, Wuhan, 430022 China; 2 Cancer Center, Union Hospital, Tongji Medical College, Huazhong University of Science and Technology, Wuhan, 430022 China; 3 Department of infectious disease, Union Hospital, Tongji Medical College, Huazhong University of Science and Technology, Wuhan, 430022 China; 4 Department of Ultrasonography, Renmin Hospital of Wuhan University, Wuhan, Hubei province 430060 China; 5 Department of Pathology, Union Hospital, Tongji Medical College, Huazhong University of Science and Technology, Wuhan, 430022 China; 6 Department of Surgical Oncology, First Affiliated Hospital of Xi’an JiaoTong University, Xi’an, 710061 China; 7 Department of Gastrointestinal Surgery, Union Hospital, Tongji Medical College, Huazhong University of Science and Technology, Wuhan, 430022 China

**Keywords:** Cancer microenvironment, Colorectal cancer

## Abstract

The relationship between systemic inflammation and tumor-associated bacteria is largely unknown in colorectal cancer (CRC). The primary aim of this study was to investigate the prognostic effects of the systemic inflammation response index (SIRI) on the survival outcomes of CRC patients who experienced surgical therapy, and the second aim was to reveal the potential association between SIRI levels and tumor-associated bacteria in CRC. We recruited a cohort of 298 CRC patients who experienced surgical resection in Wuhan Union Hospital. These patients were assigned to the low and high groups based on the cut-off value of SIRI. We utilized 1:1 propensity score matching (PSM) to reduce the potential confounding factors between the low SIRI group (*N* = 83) and the high SIRI group (*N* = 83). The total DNA of 166 paraffin-embedded tumor tissues and 24 frozen tumor tissues was extracted and amplified, and 16 S rRNA sequencing was employed to uncover the composition of microbiota between low and high SIRI groups. Survival analysis uncovered that the high SIRI cohort exhibited significantly shorter overall and disease-free survival time than low SIRI companions after PSM. The ROC analyses showed that the prediction abilities of SIRI were much higher than other serum inflammatory biomarkers for survival outcomes. The microbial richness and diversity in the low SIRI group were remarkably higher than those in the high SIRI group. At the phylum level, we found that *Proteobacteria, Synergistetes, WPS-2, Thermil, Fusobacteria* were enriched in the high SIRI group. *Cupriavidus, Thermus, Ochrobactrum, Cupriavidus, Acidovorax* were enriched in the high SIRI group at the genus level. 16 S rRNA based on frozen samples also obtained similar results. SIRI is a promising and novel prognostic biomarker among CRC sufferers who underwent surgical removal. There existed significant differences in the diversity and compositions of tumor-associated bacteria between the low and high SIRI groups.

## Introduction

Colorectal cancer (CRC) is universally acknowledged as the second cause of cancer-related death worldwide [1]. Similar to other types of malignant tumors, CRC is also characterized by tumor heterogeneity that poses great challenges to its treatment. Although the targeted therapy and immunotherapy could prolong the survival time to some extent [2], the long-term survival of the CRC population remains poor with a 5-year survival rate of nearly 60% in early staged patients [3]. Unfortunately, about half of sufferers will eventually progress to distant metastases. Despite the fact that extensive researches are related to the prognostic biomarkers of CRC patients, accurate prediction of the survival time of CRC individuals is still a tough task for oncologists [4]. Therefore, to optimize the survival prediction of the CRC population more accurately, it’s imperative to design effective prognostic biomarkers for them.

Chronic inflammation is closely related to the initiation of multiple cancers, including CRC [5]. The chronic inflammatory response can present in different tumor stages and lead to genetic modification as well as genomic instability [6]. The systemic inflammatory response could be well reflected by some serum inflammatory markers derived from complete blood counts [7]. There have been quite a few clinical investigations related to pre-treatment serum inflammatory markers which help in predicting post-operative survival of CRC patients, such as SII, PNI, ALRI, NLR and PLR. Recently, Jin et al. [8]. put forward a novel inflammation index based on peripheral neutrophils, monocytes and lymphocytes count, called the systemic inflammation response index (SIRI) in cardiovascular diseases, and exhibited talented prognostic ability in most malignant tumors [9–13]. Moreover, a recent meta-analysis included 10754 cancer patients from 38 clinical cohorts demonstrated that serum SIRI is a universal prognostic biomarker in individuals with cancer [14]. However, there is no clinical evidence indicating whether SIRI can also serve as a survival indicator to precisely predict CRC patient outcome.

The human gut microbiota includes >100 billion bacteria, viruses andparasites that maintain symbiotic interactions with the host [15]. Dysbiosis may contribute to the occurrence and progression of CRC. Colibactin, secreted by Escherichia coli, provides a prior advantage to compete with other bacteria to locate in cancerous lesions [16] and also causes DNA damage that may lead to the development of CRC [17]. Parasites are the important composition of gut microbiota. Among them, Schistosomal is the common infestation of the colorectal tract, and has been implicated in the occurrence and progression of CRC [18]. Gut eukaryotic virome is a new research area, and a recent study demonstrates that alterations in enteric virome are correlated with the progression and prognosis of CRC [19]. Increasing evidence proves that changes in the tumor-associated bacteria could affect the body’s metabolic and immune function, allowing environmental factors to initiate and promote CRC [20–22]. Therefore, modulation of tumor-associated bacteria might be one of the most promising new strategies to prevent and conquer CRC [23]. Recent researches have identified *fusobacterium nucleatum, enterococcus faecalis* as confirmed pathogens of CRC [24]. Chen et al. [24]. put forward that gut microbiota could provide some bacterial metabolites and inhibit intestinal inflammation. The dysbiosis occupies a critical role in the pathogenesis of CRC, causing initial inflammation response via modulating different inflammatory signaling pathways [25, 26]. Although quite a few microbiologists attempt to uncover the potential association between tumor-associated bacteria and chronic inflammation among CRC individuals, the relationship between the tumor-associated bacteria and serum inflammatory biomarkers as reflected by SIRI is still unknown in patients with CRC.

In the present study, we used propensity score matching (PSM) and survival analysis to investigate whether SIRI could be utilized for risk stratification among CRC individuals who experienced surgical intervention. Then, we explored the difference in tumor-associated bacteria between low and high SIRI groups based on paraffin-embedded tumor tissues and frozen tissues. As SIRI is a novel index that could reflect the systemic inflammatory response, our analysis is designed to reveal the potential association between tumor-associated bacteria and systemic inflammatory response among CRC patients.

## Materials and methods

### Cohort selection

We screened patients with CRC from Wuhan Union hospital between July 2013 and September 2017. Most of them underwent radical tumor resection, and some sufferers with advanced TNM stage experienced partial resection. The inclusion criteria: (1) The confirmed diagnosis of CRC via pathological reports; (2) Patients with intact laboratory data and follow-up information; (3) The tumor tissue is large enough for 16 S rRNA sequencing. The exclusion criteria: (1) Patients used antibiotics due to the acute bacterial infection before surgical resection; (2) Patients were complicated with systemic inflammatory disease; (3) Patients were reluctant to take part in this clinical research. Finally, 298 cases of CRC individuals who received surgical therapy were included in our research and all of them provided their informed consent to this research. Our research plan was approved by the clinical ethics committee before the initiation of this study (No. 2018-S377). In order to validate the reliability of the 16 S rRNA sequencing based on paraffin-embedded tissues, we prospectively collected the fresh tissues of 24 newly enrolled CRC individuals with the same inclusion criteria during November 2022 for 16 S rRNA sequencing analysis. For the exploration of the immune microenvironment between the low and high SIRI groups, we also collected the paraffin-embedded tissues of the 24 newly enrolled CRC individuals for subsequent immunohistochemistry assay.

### Data collection

We collected the following clinical information, such as gender, body mass index (BMI), age at diagnosis, primary site, T stage, tumor grade, N stage, TNM stage, tumor size, M stage, postoperative chemotherapy, and laboratory data (liver, renal function; inflammatory indexes; serum tumor markers) and follow-up information. The inflammatory indexes consist of SIRI, systemic immune-inflammation index (SII), prognostic nutritional index (PNI), neutrophil to lymphocyte ratio (NLR), platelets to lymphocyte ratio (PLR), lymphocyte to monocyte ratio (LMR), and aspartate aminotransferase to lymphocyte ratio index (ALRI). Serum tumor markers consist of CEA, CA125, CA724, and CA199. Overall survival (OS) is defined as the interval between the first day of surgical resection and the date of death or last visit, and disease-free survival (DFS) is defined as the interval between the first day of surgical resection and the date of any type of tumor progression, recurrent or last visit. SIRI [27] is calculated as the formula of neutrophil count * monocyte count/lymphocyte count, and other inflammatory indexes are defined according to the previous study [28]. In addition, we also collected the paraffin-embedded tumor tissues and frozen tissues for 16 S rRNA sequencing.

### PSM analysis

These patients were strictly matched with 1:1 between low and high SIRI groups via the nearest neighbor algorithm. We matched age at diagnosis, gender, BMI, TNM stage, histological grade, tumor size, tumor site, and chemotherapy to adjust for confounding indexes, and to facilitate the balanced comparison between the low SIRI and high SIRI groups. We applied an inverse probability of treatment weighting algorithm to further eliminate the potential imbalance between the low SIRI and high SIRI groups. A Cox proportional-hazards model was selected for the survival analysis by including the significant features in univariate Cox analysis. We also carried out sensitivity analyses in the primary cohort as well as the PSM cohort to further validate the conclusion of the univariate Cox analysis.

### DNA extraction and 16 S rRNA sequencing

We used the Omega Mag-Bind soil DNA kit (Omega Bio-Tek, Norcross, GA, USA) to abstract tumor-associated bacteria DNA from the selected samples. Agarose gel electrophoresis was utilized to quantitatively measure the purity of the total tumor-associated bacteria DNA. V3-V4 bacterial genome of 16 S rRNA gene was further amplified via a polymerase chain reaction. The forward primer sequence was 5'-ACTCCTACGGGAGGCAGCA-3' and the reverse primer sequence was 5'-GGACTACHVGGGTWTCTAAT-3'. The high-throughput sequencing library was constructed by using the Illumina TruSeq Nano DNA LT library prep kit (Illumina, San Diego, CA, USA).

### Analysis of the sequencing data

Quantitative Insights into Microbial Ecology2 (QIIME2) software was applied to preliminarily manage the raw sequences. Sequences with >97% similarity were automatically assigned to one operational taxonomic unit (OTU) via Uparse software. We also operate QIIME2 software to allocate the representative sequences taxonomically by the exploration of the Greengenes database (http://greengenes.secondgenome.com/). Alpha diversity, including Chao1, Shannon, Goods_coverage, Simpson, and observed spices, was measured using QIIME2 to compare the species diversity between low and high SIRI groups. Beta diversity was assessed to compare the differences in microbial community composition between low SIRI and high SIRI groups using principal-coordinate analysis (PCoA). Linear discriminant analysis (LDA) effect size (LEfSe) analysis was carried out to identify significant taxa between low and high SIRI groups at the levels of phylum and genus. The MetaCyc database and Kyoto Encyclopedia of Genes and Genomes (KEGG) database were explored to perform KEGG Orthology (KO) analysis.

### Immunohistochemistry

Four serial sections of 5 μm per paraffin block are obtained for the following immunohistochemistry staining. These sections were first baked at 60 °C and then deparaffinized in xylene and ethanol. After hydration, 3% hydrogen peroxidase was utilized to block endogenous peroxidase activity. Standard antigen retrieval was conducted via heating the sections immersed in citric acid solution (pH = 6.0) in a pressure boiler. Subsequently, these slides were incubated with the primary antibodies [CD20 (60271-1-Ig, Proteintech, 1:5000); CD4(ab133616, Abcam, 1:500), CD8(ab85792, abcam, 1:400), CD68(ab959, Abcam, 1:6000)] at 4°Covernight, and then incubated with second antibody. After 3,3'-diaminobenzidine tetrahydrochloride staining and hematoxylin counterstaining, the slides were scanned for further quantitative analysis. The density of CD4 + , CD20,CD68 and CD8 + T cells both invasive margin (IM) and in the core of the tumor (CT) were automatically calculated using ImageJ software (version 1.48). The software generally contains positive cells and a positive nucleus, and we used its ratio (positive cells/positive nucleus) to represent the expression status of four immune cells in CRC tissues.

### Statistical analysis

All the statistical analyses were performed via R software (version 3.0), Graphpad Prism 9, and SPSS 20.0. Availability of R codes involved in our analysis is available upon request. Accessibility of the SIRI threshold that may stratify the CRC patients into two gatherings with distinctive OS results was evaluated by using the X-tile software (version 3.6). Continuous data were presented as mean with standard deviation, and compared by *t*-test or nonparametric test, while categorical indexes were summarized as the frequency with percent and compared by chi-square or Fisher exact test. We plotted survival curves and compared the survival time between the low SIRI and high SIRI groups by log-rank test. ROC curves were drawn to compare the predictive ability of SIRI, SII, PNI, NLR, PLR, LMR, and ALRI for survival rates among CRC patients. The correlation between SIRI and other inflammatory biomarkers was quantified with Spearman analysis. The comparison of alpha diversity between the low SIRI and high SIRI groups using the Kruskal–Wallis test.

## Results

### Baseline features of included CRC individuals

A total of 298 CRC patients receiving surgical resection met the inclusion criteria, and were thus included in this research. Based on the optimal threshold of SIRI (1.4) measured by X-tile (Fig. S1), we divided these individuals into the low SIRI group (*N* = 192) and a high SIRI group (*N* = 106). As shown in Table 1, we found that histological grade (*P* = 0.01), tumor size (*P* = 0.005), N stage (*P* = 0.033), M stage (*P* = 0.005), TNM stage (*P* = 0.017), count of WBC (*P* < 0.0001), PLT(*P* < 0.001), serum ALB (*P* = 0.006), LDH (*P* < 0.001), CA72-4(*P* = 0.017), the death rate (*P* < 0.001) and recurrent rate (*P* < 0.001) are significantly different between the low and high SIRI groups. Hence, we used PSM analysis based on the ratio of 1:1 to balance these confounding factors between the two groups. The correlation between SIRI and clinical metrics in the PSM cohort and the weighted cohort is also shown in Table 1.

**Table 1:** Comparisons of clinical characteristics between low SIRI and high SIRI groups in the original, matched and weighted cohorts.

Features	Original cohort			Matched cohort			Weighted cohort		
	Low SIRI	High SIRI	*P*-value	Low SIRI	High SIRI	*P*-value	Low SIRI	High SIRI	*P*-value
N	192	106		83	83		85.5	86.3	
Age, years	57.2 (12.4)	55.3 (13.7)	0.226	5796 (11.3)	56.5 (13.4)	0.462	56.8 (12.5)	56.7 (13.4)	0.953
Gender, male, *n* (%)	105 (54.7)	67 (63.2)	0.193	46 (55.4)	50 (60.2)	0.637	52.5 (61.3)	53.8 (62.3)	0.880
BMI (kg/m^2^)	22.5 (2.9)	22.6 (2.9)	0.842	22.3 (2.4)	22.5 (3.0)	0.570	22.5 (2.8)	22.6 (3.0)	0.899
Primary site, *n* (%)			0.404			0.874			0.989
Left colon	103 (53.6)	53 (50.0)		43 (51.8)	45 (54.2)		45.7 (53.4)	45.8 (53.0)	
Right colon	48 (25.0)	34 (32.1)		25 (30.1)	22 (26.5)		23.7 (27.8)	24.7 (28.6)	
Rectum	41 (21.4)	19 (17.9)		15 (18.1)	16 (19.3)		16.1 (18.8)	15.9 (28.4)	
Histological grade, *n* (%)			0.010			0.871			0.975
Well differentiated	38 (19.8)	34 (32.1)		26 (31.3)	24 (28.9)		25.7 (30.1)	26.4 (30.6)	
Moderately	146 (76.0)	63 (59.4)		52 (62.7)	55 (66.3)		54.4 (63.6)	55.0 (63.7)	
Poorly differentiated	8 (4.2)	9 (8.5)		5 (6.0)	4 (4.8)		5.5 (6.4)	4.9 (5.7)	
Tumor size, *n* (%)			0.005			0.636			0.961
<2 cm	12 (6.2)	3 (2.8)		4 (4.8)	3 (3.6)		3.2 (3.8)	2.7 (3.1)	
2–5 cm	113 (58.9)	46 (43.4)		39 (47.0)	34 (41.0)		37.9 (44.3)	37.9 (43.9)	
≥5 cm	67 (34.9)	57 (53.8)		40 (48.2)	46 (55.4)		44.4 (52.0)	45.7 (52.9)	
T stage, *n* (%)			0.527			0.493			0.959
T1/2	14 (7.3)	6 (5.7)		6 (7.2)	3 (2.6)		4.8 (5.6)	4.7 (9.5)	
T3/4	178 (92.7)	100 (94.3)		77 (92.8)	80 (96.4)		80.7 (94.4)	81.6 (94.5)	
N stage, *n* (%)			0.033			0.868			0.984
N1	110 (57.3)	44 (421.5)		38 (45.8)	41 (49.4)		40.8 (47.8)	42.2 (48.9)	
N2	45 (23.4)	35 (33.0)		28 (33.7)	25 (30.1)		26.4 (30.9)	25.8 (29.9)	
N3	37 (19.3)	27 (25.5)		17 (20.5)	17 (20.5)		18.3 (21.4)	18.3 (21.1)	
M stage, *n* (%)			0.005			1.000			0.966
M0	169 (88.0)	79 (74.5)		68 (71.9)	68 (71.9)		70.6 (82.6)	71.1 (82.4)	
M1	23 (12.0)	27 (25.5)		15 (18.1)	15 (18.1)		14.9 (17.4)	15.2 (17.6)	
TNM stage, *n* (%)			0.017			0.634			0.881
Stage I/II	96 (50.0)	37 (34.9)		31 (37.3)	35 (42.2)		34.7 (40.7)	35.9 (41.6)	
Stage III/IV	96 (50.0)	69 (65.1)		52 (62.7)	48 (57.8)		50.8 (59.3)	50.4 (58.4)	
Chemotherapy, *n* (%)			0.758			0.532			0.899
No	81 (42.2)	42 (39.6)		39 (47.0)	34 (41.0)		36.4 (42.6)	36 (41.8)	
Yes	111 (57.8)	64 (60.4)		44 (53.0)	49 (59.0)		49.1 (57.4)	50.3 (58.2)	
Post radiotherapy, *n* (%)			0.352			0.212			0.932
No	176 (91.7)	101 (95.3)		82 (98.8)	78 (94.0)		81.8 (95.6)	82.3 (95.4)	
Yes	16 (8.3)	5 (4.7)		1 (1.2)	5 (6.0)		3.7 (4.4)	4.0 (4.6)	
Laboratory results									
WBC, ×10^9^/L	5.4 (2.0)	8.1 (3.1)	<0.001	5.3 (1.4)	8.1 (3.2)	<0.001	5.5 (1.5)	8.1 (2.3)	<0.001
HGB, g/dL	111.1 (26.0)	109.6 (24.2)	0.621	113.1 (23.0)	107.9 (25.3)	0.174	112.7 (24.9)	108.0 (24.7)	0.150
PLT, ×10^9^/L	234.5 (81.1)	276.4 (90.8)	<0.001	237.0 (97.5)	276.0 (91.5)	0.009	237.2 (89.0)	277.7 (92.6)	0.001
Albumin, g/L	39.6 (4.5)	38.0 (5.5)	0.006	39.2 (4.5)	38.0 (5.6)	0.120	39.3 (4.7)	37.7 (5.5)	0.017
TBIL, μmol/L	10.9 (3.5)	10.9 (3.9)	0.991	10.6 (3.1)	10.7 (3.1)	0.890	10.9 (5.2)	10.7 (5.0)	0.800
Creatinine, umol/L	71.5 (16.8)	73.4 (20.6)	0.399	71.7 (14.4)	73.7 (20.2)	0.463	72.1 (14.8)	72.8 (20.2)	0.769
BUN, mmol/L	4.9 (1.5)	5.0 (1.9)	0.530	4.8 (1.5)	5.1 (2.1)	0.340	5.0 (1.5)	5.1 (1.9)	0.629
LDH, U/L	179.9 (46.9)	218.4 (81.8)	<0.001	178.5 (37.4)	209.5 (96.1)	0.013	180.5 (52.4)	213.9 (90.8)	0.011
CEA, μg/L	25.4 (12.2)	224.5 (47.1)	0.062	23.4 (6.3)	233.2 (55.0)	0.251	29.3 (7.6)	241.8 (58.6)	0.224
CA19-9, U/mL	94.6 (59.6)	174.4 (93.2)	0.051	87.4 (54.4)	141.5 (78.4)	0.332	121.8 (62.0)	168.8 (72.0)	0.411
CA125, U/mL	21.0 (9.6)	38.0 (18.2)	0.129	25.8 (10.3)	30.1 (11.1)	0.629	19.9 (9.7)	30.0 (11.2)	0.040
CA72-4, U/mL	8.0 (6.5)	15.4 (15.4)	0.017	9.8 (7.0)	10.1 (6.5)	0.921	9.3 (6.0)	12.9 (11.9)	0.298
OS months	19.2 (9.1)	18.3 (9.8)	0.479	18.2 (9.0)	19.9 (10.7)	0.291	18.7 (9.3)	19.0 (10.9)	0.824
DFS months	18.8 (8.3)	16.8 (8.1)	0.120	17.7 (9.0)	18.7 (10.2)	0.565	18.1 (9.2)	18.0 (9.3)	0.907
Death, *n* (%)	18 (9.4)	31 (29.2)	<0.001	12 (14.5)	24 (28.9)	0.024	11.7 (13.7)	23.3 (26.9)	0.018
Recurrence, *n* (%)	26 (13.5)	43 (40.6)	<0.001	14 (16.9)	33 (39.8)	0.003	16.2 (18.9)	31.0 (35.9)	0.006

*SIRI* systemic inflammation response index, *BMI* body mass index, *WBC* white blood cells, *HGB* hemoglobin, *PLT* platelets, *TBIL* total bilirubin, *BUN* blood urea nitrogen, *LDH* lactate dehydrogenase, *CEA* carcinoma embryonic antigen, *CA19-9* carbohydrate antigen 19-9, *OS* overall survival, *DFS* disease free survival.

**Fig. 1:**
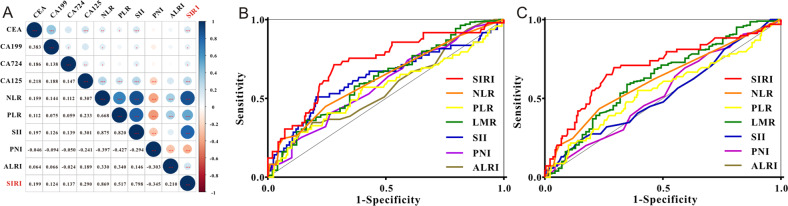
Clinical correlation and predictive ability of serum inflammatory biomarkers. **A** The relationships between SIRI with other serum inflammatory biomarkers and serum tumor biomarkers. **B** ROC curves of serum inflammatory biomarkers for the prediction of overall survival among colorectal cancer patients. **C** ROC curves of serum inflammatory biomarkers for the prediction of disease-free survival in individuals with CRC.

In the crude cohort, we assessed the correlation between SIRI and other common inflammatory indexes, such as SII, NLR, PLR, PNI, and ALRI. As shown in Fig. 1A, we discovered that SIRI exhibited a positive correlation with SII (*r* = 0.798), NLR (*r* = 0.869), and PLR (*r* = 0.517), while SIRI exhibited a negative association with PNI (*r* = −0.345). As for serum tumor markers, we found a positive relationship (*r* = 0.290) between SIRI and serum CA125, while SIRI showed a weak association with other serum tumor markers, such as CEA, CA199, and CA724. Then we used the ROC analysis to measure the predictive accuracy of common inflammatory biomarkers. Preoperative SIRI showed better AUC not only for the prediction of OS rate (Fig. 1B) but also for the DFS rate (Fig. 1C) among operative CRC individuals. The detailed comparison of each inflammatory index is listed in Table S1.

### Prognostic value of SIRI in CRC patients after PSM

**Fig. 2:**
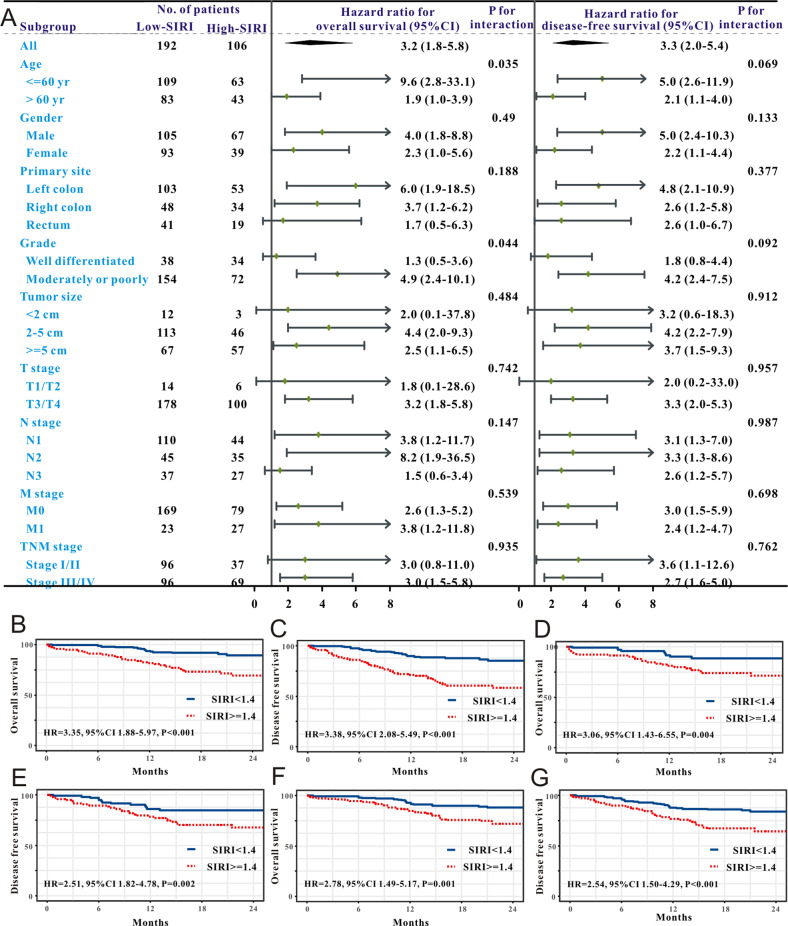
Subgroup analysis and survival analysis of SIRI in individuals with CRC. **A** The forest plot revealed the results of subgroup analysis for overall survival and disease-free survival. Kaplan–Meier plots of survival outcomes based on low and high SIRI groups in the crude cohort (**B**, **C**), PSM cohort (**D**, **E**) and weighted cohort (**F**, **G**).

In the crude CRC cohort, we used subgroup analysis to make sure whether the level of SIRI is a potent factor that independently affects the survival of CRC patients who received surgical removal. As exhibited in the forest plot, a strong relationship between high SIRI and less favorable OS existed in most subgroups (Fig. 2A), such as age, gender, and M stage. Similarly, a strong correlation between high SIRI and less favorable DFS existed in most subgroups (Fig. 2A), such as age, gender, primary site, N stage, M stage, and TNM stage.

Survival analysis was executed to assess the significance of SIRI in the stratification of CRC individuals with different survival risks. In the crude population, HR showed that a high SIRI group was correlated with a less favorable OS rate (HR = 3.21, 95%CI:1.79-5.15, *P* < 0.0001. Figure 2B) and DFS rate (HR = 3.31, 95%CI:2.03-5.38, *P* < 0.0001). Figure 2C among CRC individuals. This strong association also existed in the PSM population (Fig. 2D, E) and weighted cohort (Fig. 2F, G).

We adopted the univariate Cox model to explore the effects of SIRI on survival outcomes (OS & DFS) in the whole population, PSM population, and weighted cohort. As listed in Table 2, we noticed that high SIRI is the risk factor for inferior survival outcomes among CRC individuals. We also employed sensitivity analysis to confirm the positive relationship between SIRI and prognosis. After the adjustment for potential covariates in the three models, this association remained significant (*P* < 0.05).

**Table 2:** Results of clinical outcomes and sensitivity analysis.

Clinical models	Overall survival		Disease-free survival	
	HR (95%CI)	*P*-value	HR (95%CI)	*P*-value
Cox proportional hazards model	3.21 (1.79-5.75)	<0.001	3.31 (2.03-5.38)	<0.001
Cox proportional hazards model with adjust I	3.22 (1.79-5.76)	<0.001	3.35 (2.05-5.45)	<0.001
Cox proportional hazards model with adjust II	2.84 (1.48-5.46)	0.002	2.53 (1.47-4.36)	<0.001
Cox proportional hazards model with adjust III	2.39 (1.15-4.99)	0.011	2.26 (1.23-4.15)	0.009
Propensity score matching	2.19 (1.10-4.24)	0.026	2.28 (1.18-4.73)	0.016
Propensity score matching with adjust I	2.21 (1.79-5.09)	0.009	2.01 (1.31-3.62)	0.011
Propensity score matching with adjust II	2.79 (1.39-6.04)	0.012	2.86 (1.89-5.78)	0.004
Propensity score matching with adjust III	2.33 (1.26-6.81)	0.023	2.64 (1.41-6.20)	0.018
Propensity score IPW	2.47 (1.17-5.21)	0.018	2.12 (1.11-3.48)	0.021
Propensity score IPW with adjust I	2.56 (1.20-5.45)	0.015	1.96 (1.39-4.01)	0.001
Propensity score IPW with adjust II	2.49 (1.35-4.60)	0.004	2.30 (1.32-3.99)	0.003
Propensity score IPW with adjust III	2.19 (1.18-4.86)	0.0029	2.15 (1.14-4.06)	0.019

Adjust I model adjusted for age, gender, *BMI* Adjust II model adjusted for adjust I model plus differentiation, TNM stage, T stage, N stage, M stage, primary site, tumor size, adjuvant chemotherapy, post radiotherapy. Adjust III model adjusted for adjust II model plus laboratory results.

### Composition of tumor-associated bacteria between low and high SIRI groups

**Fig. 3:**
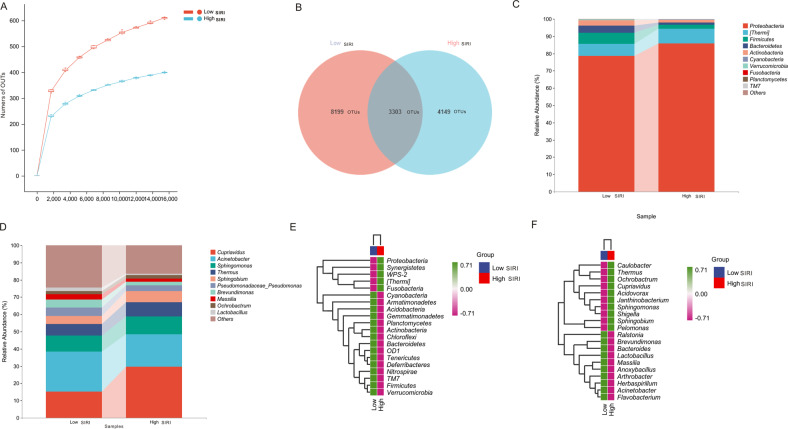
Rarefaction curve and gut microbiota composition between low and high SIRI groups. **A** Rarefaction curve. **B** The Venn plots reveal the unique and common taxa between low and high SIRI groups. Compositions of gut microbial taxonomic at the phylum (**C**) and genus (**D**) levels. Up-regulated and downregulated microbial taxonomic between low and high SIRI groups at the phylum (**E**) and genus (**F**) levels.

We drew the rarefaction curve to assess whether the sample size is enough for our analysis. As shown in Fig. 3A, the end of the curve lines for the low SIRI group and high group tend to be flat, implicating that the number of 16 S rRNA sequencing is almost reasonable. The rarefaction curve shows that more CRC cases will only add a few new OTUs, indicating the CRC cases are enough to cover most gut bacteria. A total of 15651 OTUs were identified in our analysis, including 11502 in the low SIRI group, 7452 in the high SIRI group, and 3303 OTUs in both low and high groups (Fig. 3B), indicating that OTUs were significantly more in low SIRI group than that in high SIRI groups. Microbial taxon assignment was used to compare the composition of tumor-associated bacteria between low and high SIRI groups at the levels of phylum and genus. As shown in Fig. 3C, the most abundant phyla between low and high SIRI groups at the phylum level were *Proteobacteria, Thermi, Firmicutes, Bacteroidetes, Actinobacteria*, *Verrucomicrobia, Fusobacteria, Planctomycetes* and *TM7*. We noticed that the abundance of *Fusobacteria* was significantly richer in the high SIRI group than that in the low SIRI group. At the genus level (Fig. 3D), the most abundant bacteria between low and high SIRI groups were *Cupriavidus, Acinetobacter, Sphingomonas, Thermus, Sphingobium, Pseudomonadaceae_Pseudomonas, Brevundimonas, Massilia, Ochrobactrum* and *Lactobacillus*. We could observe that the abundance of *Acinetobacter* was remarkably richer in *the* high SIRI group than that in the low SIRI group. Then, we further identified the most significant microbiota between the low and high SIRI groups. At the phylum level, we found that *Proteobacteria, Synergistetes, WPS-2, Thermil, Fusobacteria* were enriched in patients with the high SIRI group, while *Cyanobacteria, Armatimonadetes, Acidobacteria, Gemmatimonadetes, Planctomycetes, Actinobacteria, Chloroflesxi, OD1, Tenericutes, Deferribacteres, Nitrospire, TM7, Fimicutes and Verrucomicrobia* were enriched in low SIRI group (Fig. 3E). At the genus level, the abundance of *Cupriavidus, Thermus, Ochrobactrum, Cupriavidus, Acidovorax, Janthinobacterium, Sphingomonas, Sphingobium, Shigella, Sphingobium* and *Pelomonas* were up-regulated in high SIRI group, while *Ralstonia, Brevundimonas, Bacteroides, Lactobacillus, Massilia, Anoxybacillus, Arthrobacter, Herbaspirillum, Acinetobacter* and *Flavobacterium*, were up-regulated in low SIRI group (Fig. 3F).

**Fig. 4:**
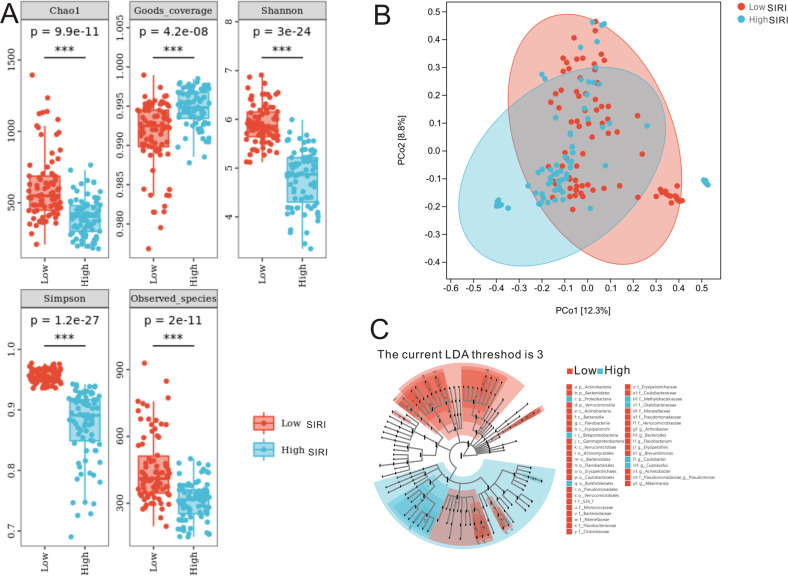
Diversity of microbiota and LEfSe analyses of the low and high SIRI groups. **A** Alpha diversity reveals that species richness was different between the two groups. **B** Beta diversity analysis revealed by PCoA. **C** LEfSe analysis revealed that there was a remarkable difference in species diversity between the two groups.

As alpha diversity is the general reflection of species richness, we applied this analysis to determine the richness of the tumor-associated bacteria between low and high SIRI groups. As listed in Fig. 4A, indexes of Chao 1(*P* = 9.9e-11), Goods coverage (*P* = 4.2e-8), Shannon (*P* = 3e-24), Simpson (*P* = 1.2e-27) and observed spices (*P* = 2e-11) were remarkably different between the low and high SIRI groups, indicating that species richness was significantly richer in low SIRI group than that in high SIRI group. Based on principal-coordinate analysis (PCoA), we found that PCo1 is 12.3% and PCo2 is 8.8% (Fig. 4B). LEfSe analysis revealed that there was a remarkable difference in species diversity between the two groups (Fig. 4C). When the LDA threshold was set at 3, a total of 42 species were identified in the low SIRI and high SIRI groups. Seven species were enriched in the high SIRI group, and 35 species were enriched in the low SIRI group. The 16 S rRNA sequencing results of CRC tissues were analyzed by KEGG and KEGG Orthology analyses between SIRI low and high groups. Figure S2 shows the relative abundances of functional pathways. The top 5 pathways of biosynthesis were amino acid biosynthesis; cofactor, prosthetic group, electron carrier, vitamin biosynthesis; nucleoside and nucleotide biosynthesis; fatty acid and lipid biosynthesis, and carbohydrate biosynthesis. In addition, the KO analysis uncovered eight significant metabolic pathways between SIRI low and high groups (Table S2).

### Validation with frozen CRC tissues

We also used the 24 cases of frozen tissues from newly enrolled CRC individuals for 16 S rRNA sequencing. The most abundant phyla between the low SIRI group and high SIRI group at the phylum level (Figure S3A) are *Proteobacteria, Thermi, Firmicutes, Actinobacteria, Bacteroidetes, Cyanobacteria* and *TM7*. At the genus level, the most abundant bacteria between low and high SIRI groups were *Cupriavidus, Acinetobacter, Sphingomonas, Thermus, Psesudomonadaceae_Pseudomonas, Brevundimonas* and *Sphingobium* (Figure S3B). Hence, the abundance between the low SIRI group and high SIRI group both at the phylum and genus levels based on frozen tissues is similar to the abundance based on paraffin tissue samples. Moreover, alpha diversity is also different between the low SIRI group and the high SIRI group based on fresh tissues(Figure S3C). Results of LEfSe analysis showed that there is a significant difference in species diversity between the low SIRI group and the high SIRI group based on frozen tissues (Figure S3D).

### Comparison of immune cells in the low and high SIRI groups

**Fig. 5:**
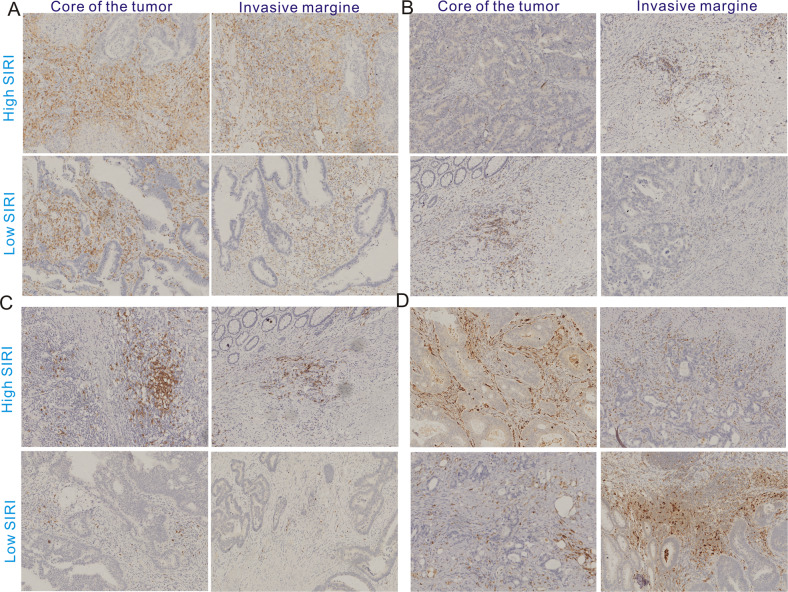
The representative staining images of immune cells in the low and high SIRI groups. **A** CD4 + T cells; **B** CD8 + T cells; **C** CD20 + B cells; **D** CD68 + macrophages.

We used the immunohistochemistry assay to determine the expression of four immune cells in the low and high SIRI groups. The density of CD4 + T cells regarding the ratio of positive cells to the positive nucleus in the IM and CT seem to be higher in the high SIRI group than that in the low SIRI group (Fig. S4A), but the difference between the low SIRI group and the high SIRI group is statistically insignificant (Fig. S4B). Moreover, CD8 + T cells (Fig. S4C, D), CD20 B cells (Fig. S4E, F) and macrophages (Fig. 4G, H) exhibited no significant difference neither in IM nor CT of CRC tissues. The representative staining figures of CD4 T cells, CD8 T cells, CD20 B cells and macrophages are listed in Fig. 5A–D. The insignificant difference in immune cells between the low SIRI and high SIRI is more likely due to the small sample size of newly enrolled CRC patients.

## Discussion

Large metagenomic evidence highlights an essential role of the intestinal microbiota in chronic gut inflammation and CRC [29]. Tumor-associated bacteria plays a pivotal role in shaping inflammatory environments, which could enhance the tumor growth and metastasis of CRC [30]. Intestinal dysbiosis will lead to the decrease of beneficial bacteria-derived metabolites, enhanced release of toxic metabolites secreted by bacteria, and the disruption of the epithelial barrier, which could incur the aberrant activation of the immune response with chronic inflammation and thus promote the progression of CRC [31]. Hence, gaining deep insights into the correlation between tumor-associated bacteria and systemic inflammation in CRC is of great significance.

In the present study, we initially explored the prognostic significance of SIRI for stratifying CRC individuals with different survival risks, and we observed that high SIRI was not only connected with the worse OS but also linked to poorer DFS in CRC sufferers. ROC curves demonstrated that SIRI possessed a superior predictive ability for the survival rate of CRC patients to other common inflammatory biomarkers, such as SII, PNI, NLR, and PLR. Then, we especially compared the composition of gut microbiota between low SIRI and high SIRI patients via 16 S rRNA gene sequencing, and we noticed that there existed significant differences in the diversity and compositions of tumor-associated bacteria between the low and high SIRI groups, indicating that high levels of inflammation reduced the diversity of gut microbiota in CRC patients.

A clinical trial explored the correlation between SIRI and pathological complete response in patients with 241 cases of breast cancer receiving chemotherapy and concluded that serum SIRI could predict pathological complete response in breast cancer individuals receiving chemotherapy [12]. This conclusion was proved by another clinical trial containing 262 cases of breast cancer individuals [32]. Sun et al. [9] demonstrated that a high level of SIRI (≥0.89) is an independent predictor of worse prognosis among gallbladder cancer patients. Huang et al. [33] found that SIRI is useful in helping the differentiation of malignant and benign ovarian tumors, while this study did not assess the prognostic value of SIRI among ovarian tumor patients. Moreover, a recent study [34] investigated the prognostic influence of the SIRI on the survival outcomes of lung cancer patients receiving concurrent chemoradiotherapy and concluded that a high level of SIRI could independently affect the survival outcomes among those patients. However, the prognostic influence of SIRI on patients with CRC is still unknown. Our study focused on the clinical and prognostic significance of SIRI among CRC individuals, and we found that a high level of SIRI was correlated with less favorable survival outcomes of CRC patients not only in the entire cohort but also in the PSM cohort.

Recent studies have revealed that tumor-associated bacteria is pervasive among malignant tumors and a significant factor in cancer immunotherapy [35–37]. Yu et al. [38] collected fecal samples from 49 matched healthy individuals, 23 cases of primary gastric cancer, 26 metastatic gastric cancer patients, and the results of 16 S rRNA gene sequencing revealed that Streptococcus alteration was significantly correlated with liver metastasis of gastric cancer. Erick et al. [39] used 16 S rRNA gene sequencing to compare the composition of tumor-associated bacteria in pancreatic cancer patients with different survival times, and found that higher alpha diversity of tumor tissue is more likely to correlate with longer survival time. Another study applied The Microbe Identification Microarray to test for the presence of 272 bacterial species from 333 upper digestive tract tissues and found that decreased microbial abundance in the upper digestive tract was closely associated with both cancer-predisposing states [40]. Although most of these studies included similar sample sizes between different groups, the two groups are still not balanced regarding baseline features, which will cause bias in their study conclusions. As our current study was a retrospective cohort analysis, we determined propensity scores for low and high SIRI groups to adjust for confounding variables. We believe that PSM analysis will facilitate well-balanced comparability between the low and high SIRI groups. Gut microbiota is easily affected by many clinical factors, such as race, age, gender, and TNM stage, we employed a 1:1 PSM analysis to balance low and high SIRI groups, and we found that high levels of SIRI correlated well with the worse survival outcomes in CRC individuals experiencing surgical resection. After PSM, compositions of tumor-associated bacteria between low SIRI and high SIRI were significantly different.

Dysbiosis promotes chronic inflammation and carcinogenesis, and a high level of systemic inflammation is correlated with worse survival outcomes in CRC individuals. However, the potential relationship between systemic inflammation and gut microbiota is largely unknown in CRC. Bacteroidetes are reported to be closely linked to chronic intestinal inflammation [41]. Moreover, an increased abundance of Fusobacterium was detected in the intestinal tracts of individuals with CRC [42], and F. nucleatum subsp. polymorphism secrets outer membrane vesicles, which could produce NF-κB and TLR4 to activate pro-inflammatory pathways [43]. Our analysis utilized PSM to balance the low and high SIRI groups, to identify the most significant bacteria between the two groups in CRC individuals. At the phylum level, we noticed that *Fusobacteris* was enriched in the high SIRI group, indicating *Fusobacteris* plays a roinflammatory role in CRC occurrence and progression. We found that cupriavidus, acinetobacter, and sphingomomas are the top three bacteria among CRC patients with high SIRI. An experimental study reveal that a high-fat diet produced a pro-inflammatory microenvironment characterized by the increased abundance of sphingomomas [44]. Our study also pointed out that the abundance of sphingomomas was enriched in high inflammatory tissues in CRC.

Our study not only includes noteworthy strengths but also contains two limitations. We investigated the characteristic differences in microbiota profiles between low SIRI (*N* = 83) and high SIRI (*N* = 83) groups in CRC with PSM analysis. However, two obvious drawbacks also existed in our analysis. First, this was a retrospective analysis and we could only collect paraffin tissue samples, which may somewhat affect the composition of gut microbiota. Although we validated the reliability of 16 S rRNA sequence with frozen CRC tissues, the sample size of newly collected tissues is small due to the limited time. Next, the exact mechanism of how inflammation status reflected by high SIRI affected changes in tumor-associated bacteria composition in CRC is still unknown, and metagenomics along with metabolomics might be deductive to solve this clinical issue.

## Conclusion

Our results in the PSM cohort of 166 cases of CRC patients treated with surgical removal showed that a high level of baseline SIRI was a robust biomarker to predict remarkably worse survival outcomes. Moreover, we detected significant differences in the compositions of tumor-associated bacteria between low and high SIRI groups and found that the diversity of microbiota in the low SIRI group was significantly richer than that in the high SIRI group. Our study roughly revealed the potential correlation between systemic inflammation and tumor-associated bacteria in CRC patients.

## Supplementary information

Supplementaary Material

Supplementary Figure and Table legends

Reproducibility Checklist

## Data Availability

The original data analyzed in the present study are available from the corresponding author on reasonable request.
