# Supplementary material for: Levels of systemic inflammation response index are correlated with tumor-associated bacteria in colorectal cancer
Source: Cell Death Dis. 2023 Jan 30;14(1):69. doi: 10.1038/s41419-023-05602-9 (PMC9886998; doi:10.1038/s41419-023-05602-9)
Supplement: Supplementary file 1 — Supplementaary Material [file 41419_2023_5602_MOESM1_ESM.doc]

**Supplementary information**

**Supplementary Figures**



**Supplementary Figure 1**. The best cut-off threshold of SIRI is 1.4, which was determined by X-tile software (version 3.6).


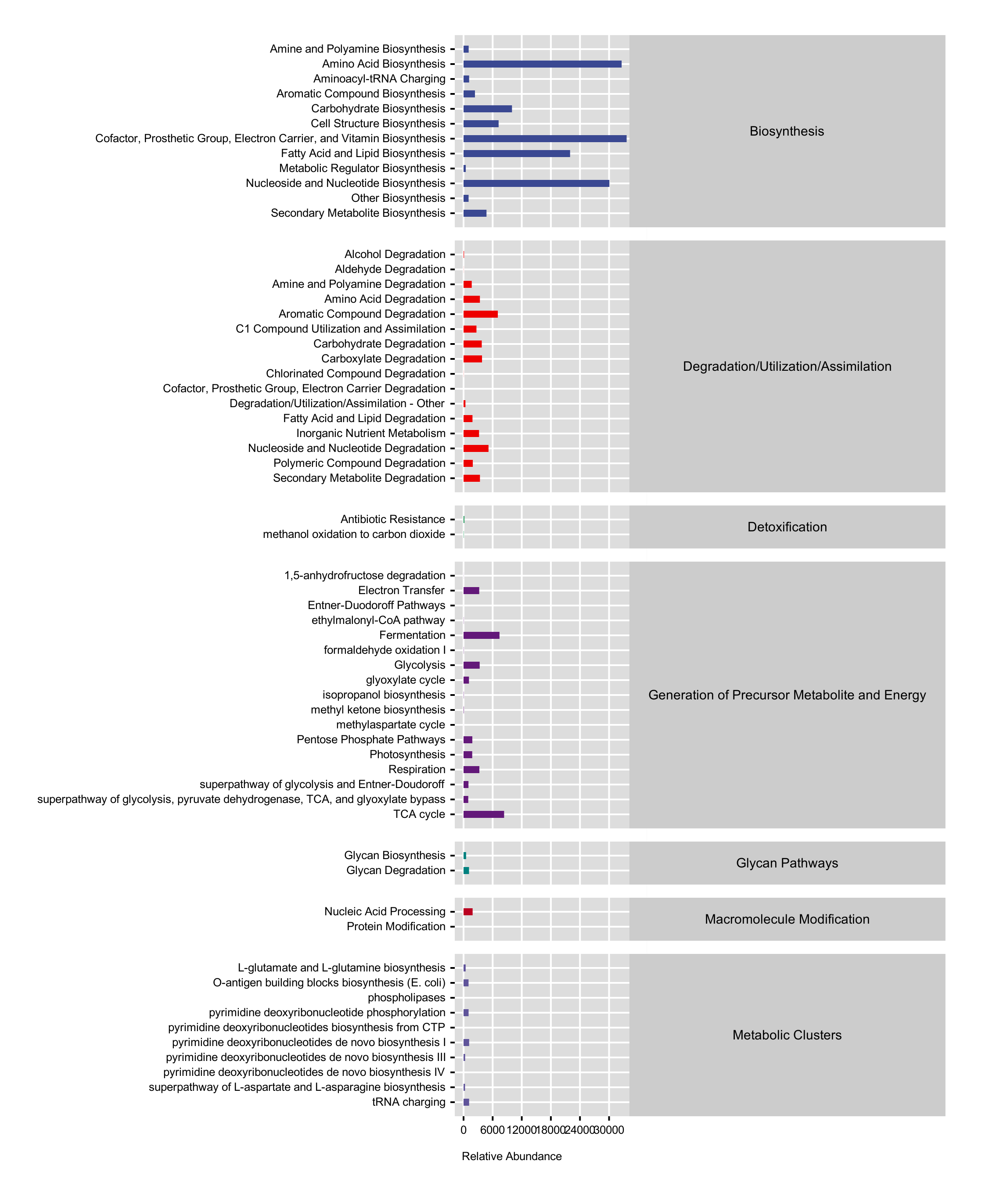


**Supplementary Figure 2**. KEGG pathways for the low and high SIRI groups revealed by 16S rRNA sequencing.


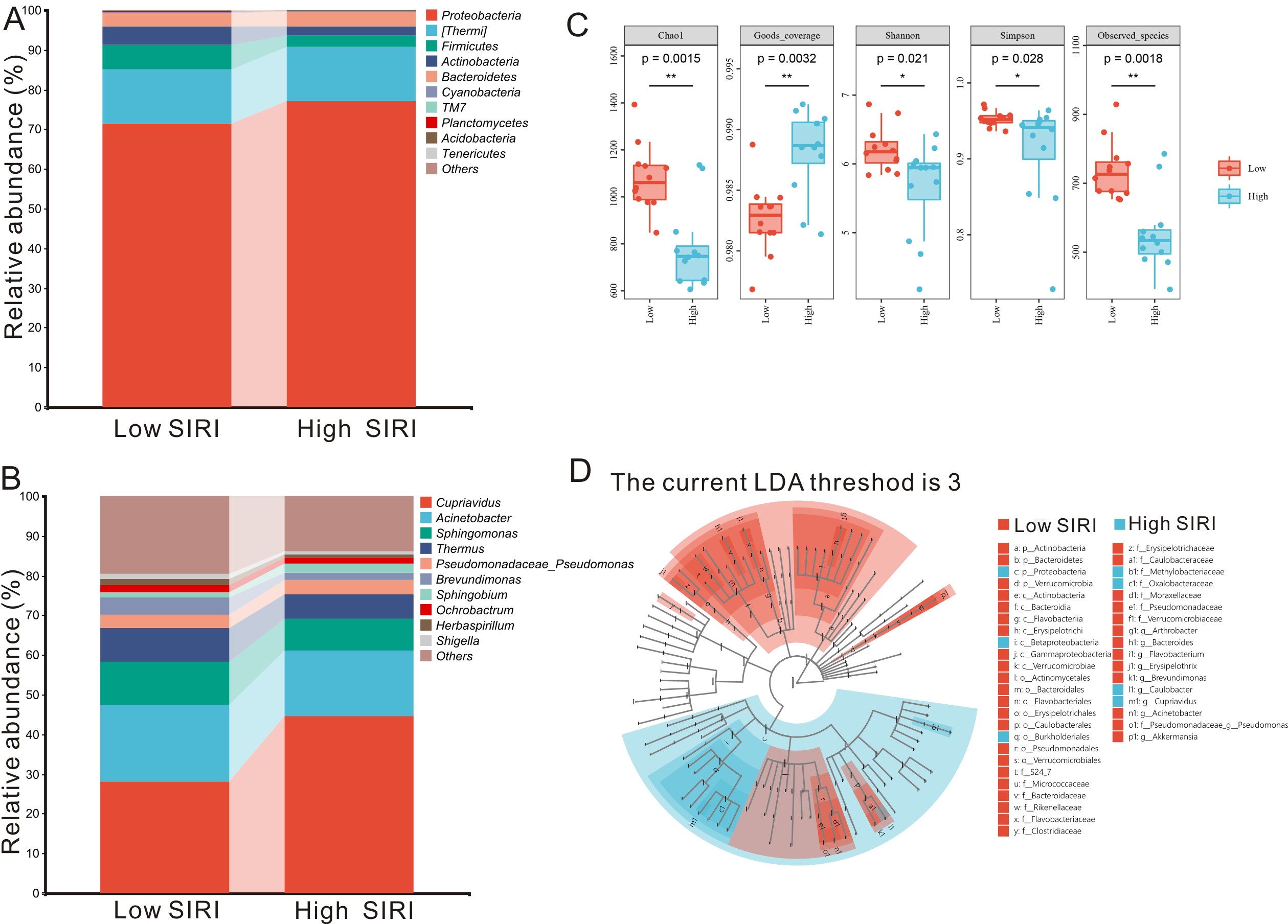


**Supplementary Figure 3**. Composition of tumor-associated bacteria and alpha diversity between low SIRI group and high SIRI group based on frozen tissues. Compositions of tumor-associated bacteria taxonomic at the phylum level (**A**) and genus (**B**) level. Alpha diversity exhibited the different species richness between the low and high SIRI groups (**C**). LEfSe analysis revealed the remarkable difference in species diversity between the low and high SIRI groups (**D**).





**Supplementary Figure 4**.quantitative analysis of four immune cells in the low and high SIRI groups via ImageJ software. **A**. Ratio of CD4+ T cells in the core of tumor. **B**. Ratio of CD4+ T cells in the invasive margin. **C**. Ratio of CD8+ T cells in the core of tumor. **D**. Ratio of CD8+ T cells in the invasive margin. **E**. Ratio of CD20+ B cells in the core of tumor. **F**. Ratio of CD20+ B cells in the invasive margin. **G**.Ratio of CD68+ macrophages in the core of tumor. **H**. Ratio of CD68+ macrophages in the invasive margin.

**Supplementary Tables**

**Supplementary Table 1.** **Receiver operating curve analysis of serum inflammatory indexes**

| Variables | Sensitivity | Specificity | AUC (95%CI) | P value |
| --- | --- | --- | --- | --- |
| For overall survival |  |  |  |  |
| SIRI | 71.4 | 71.9 | 0.725 (0.671-0.775) |  |
| NLR | 44.9 | 75.5 | 0.627 (0.570-0.683) | 0.0092 |
| PLR | 57.1 | 59.8 | 0.558 (0.500-0.616) | 0.0006 |
| LMR | 63.3 | 57.8 | 0.623 (0.566-0.679) | 0.0027 |
| SII | 51.0 | 79.5 | 0.632 (0.574-0.686) | 0.0284 |
| PNI | 40.8 | 74.3 | 0.593 (0.535-0.649) | 0.0068 |
| ALRI | 34.7 | 81.1 | 0.565 (0.506-0.622) | 0.0009 |
| For disease free survival |  |  |  |  |
| SIRI | 69.6 | 70.7 | 0.699 (0.643-0.750) |  |
| NLR | 43.5 | 76.9 | 0.616 (0.558-0.671) | 0.0113 |
| PLR | 55.1 | 60.7 | 0.549 (0.491-0.607) | 0.0010 |
| LMR | 65.2 | 59.0 | 0.625 (0.567-0.680) | 0.0230 |
| SII | 52.2 | 73.8 | 0.624 (0.566-0.679) | 0.0423 |
| PNI | 40.6 | 69.4 | 0.535 (0.476-0.592) | 0.0006 |
| ALRI | 23.2 | 86.0 | 0.527 (0.469-0.585) | 0.0002 |

SIRI, systemic inflammation response index, NLR, neutrophil to lymphocyte ratio, PLR, platelets to lymphocyte ratio, LMR, lymphocyte to monocyte ratio, SII, systemic immune-inflammation index, PNI, prognostic nutritional index, ALRI, aspartate aminotransferase to lymphocyte ratio index, AUC, area under the receiver operating curve, 95%CI, 95% confidence interval.

P value was calculated by the comparison of SIRI and other inflammation indexes.

**Supplementary Table 2.** KEGG Orthology metabolic pathways in low and high SIRI groups

| pathway | Description | logFC | SE | P values | adjPvalues |
| --- | --- | --- | --- | --- | --- |
| PWY-6107 | Chlorosalicylate degradation | 0.6647 | 0.1758 | 0.0001563 | 0.008735 |
| PWY-5529 | Superpathway of bacteriochlorophyll a biosynthesis | -0.7563 | 0.1407 | 7.70E-08 | 0.00001147 |
| CHLOROPHYLL-SYN | Chlorophyllide a biosynthesis I (aerobic, light-dependent) | -0.7626 | 0.1445 | 1.32E-07 | 0.00001474 |
| PWY-7024 | Superpathway of the 3-hydroxypropanoate cycle | -0.8573 | 0.193 | 0.000008961 | 0.0006676 |
| PWY-6731 | Starch degradation III | -0.8748 | 0.1539 | 1.32E-08 | 0.000002956 |
| PWY-5743 | 3-hydroxypropanoate cycle | -0.8867 | 0.2049 | 0.00001514 | 0.000967 |
| PWY-5744 | Glyoxylate assimilation | -0.9015 | 0.199 | 0.000005906 | 0.000528 |
| PWY-3941 | beta;-alanine biosynthesis II | -0.9972 | 0.1572 | 2.23E-10 | 9.96E-08 |
