# Supplementary material for: Levels of systemic inflammation response index are correlated with tumor-associated bacteria in colorectal cancer
Source: Cell Death Dis. 2023 Jan 30;14(1):69. doi: 10.1038/s41419-023-05602-9 (PMC9886998; doi:10.1038/s41419-023-05602-9)
Supplement: Supplementary file 2 — Supplementary Figure and Table legends [file 41419_2023_5602_MOESM2_ESM.docx]

**Supplementary Figure and Table legends**

**Supplementary Figure 1**. The best cut-off threshold of SIRI is 1.4, which was determined by X-tile software (version 3.6).

**Supplementary Figure 2**. KEGG pathways for the low and high SIRI groups were revealed by 16S rRNA sequencing.

**Supplementary Figure 3**. Composition of tumor-associated bacteria and alpha diversity between low SIRI group and high SIRI group based on frozen tissues. Compositions of tumor-associated bacteria taxonomic at the phylum level (**A**) and genus (**B**) level. Alpha diversity exhibited the different species richness between the low and high SIRI groups (**C**). LEfSe analysis revealed the remarkable difference in species diversity between the low and high SIRI groups (**D**).

**Supplementary Figure 4**. Quantitative analysis of four immune cells in the low and high SIRI groups via ImageJ software. **A**. Ratio of CD4+ T cells in the core of the tumor. **B**. Ratio of CD4+ T cells in the invasive margin. **C**. Ratio of CD8+ T cells in the core of the tumor. **D**. Ratio of CD8+ T cells in the invasive margin. **E**. Ratio of CD20+ B cells in the core of the tumor. **F**. Ratio of CD20+ B cells in the invasive margin. **G**. Ratio of CD68+ macrophages in the core of the tumor. **H**. Ratio of CD68+ macrophages in the invasive margin.

**Supplementary Tables**

**Supplementary Table 1.** Receiver operating curve analysis of serum inflammatory indexes.

**Supplementary Table 2.** KEGG Orthology metabolic pathways in low and high SIRI groups.
